# Development and expert consensus on an action protocol for multidisciplinary vascular access teams

**DOI:** 10.1515/med-2026-1405

**Published:** 2026-05-11

**Authors:** Filipa Isabel Martins Santos, Vítor Rodrigues, Carlos Almeida

**Affiliations:** School of Health, University of Trás-os-Montes e Alto Douro, Vila Real, Portugal; RISE-Health: Health Research and Innovation, Research Center in Sports Sciences, Health Sciences and Human Development, CIDESD, Clinical Academic Center of Trás-os-Montes and Alto Douro, CACTMAD, School of Health, University of Trás-os-Montes e Alto Douro, Vila Real, Portugal

**Keywords:** multidisciplinary vascular access team, protocol, ultrasound-guided access, delphi study, vascular access management

## Abstract

**Objectives:**

The implementation of specialized multidisciplinary vascular access teams (VATs), composed of healthcare professionals with advanced expertise, has been recommended to ensure best practices in the insertion, management, and monitoring of vascular access devices. The aim is to standardize the intervention of a multidisciplinary vascular access team, through the construction of an action protocol.

**Methods:**

This mixed-method study used an exploratory, descriptive qualitative approach followed by a quantitative Delphi technique to reach expert consensus. Twenty healthcare professionals participated in the qualitative phase, while twenty experts, all trained in ultrasound-guided vascular access, were involved in the quantitative phase.

**Results:**

The qualitative analysis revealed the need for a structured protocol addressing device selection, patient assessment, and activation criteria. The quantitative phase achieved 100 % consensus on the proposed interventions, leading to the construction of a validated flowchart for VAT actions.

**Conclusions:**

A consensus-based protocol was successfully developed, integrating evidence-based recommendations and expert consensus. This protocol can improve clinical outcomes, patient satisfaction, and resource optimization and can be adapted for implementation in other healthcare institutions.

## Introduction

Millions of vascular catheters are placed annually in healthcare services, with over 90 % of hospitalized patients requiring venous access. Despite their importance, catheter placement often faces challenges, particularly in patients with difficult intravenous access (DIVA). Up to 26 % of adults may experience multiple failed attempts, increasing complications and healthcare costs [1], [2], [3], [4], [5], [6], [7].

**Figure 1:**
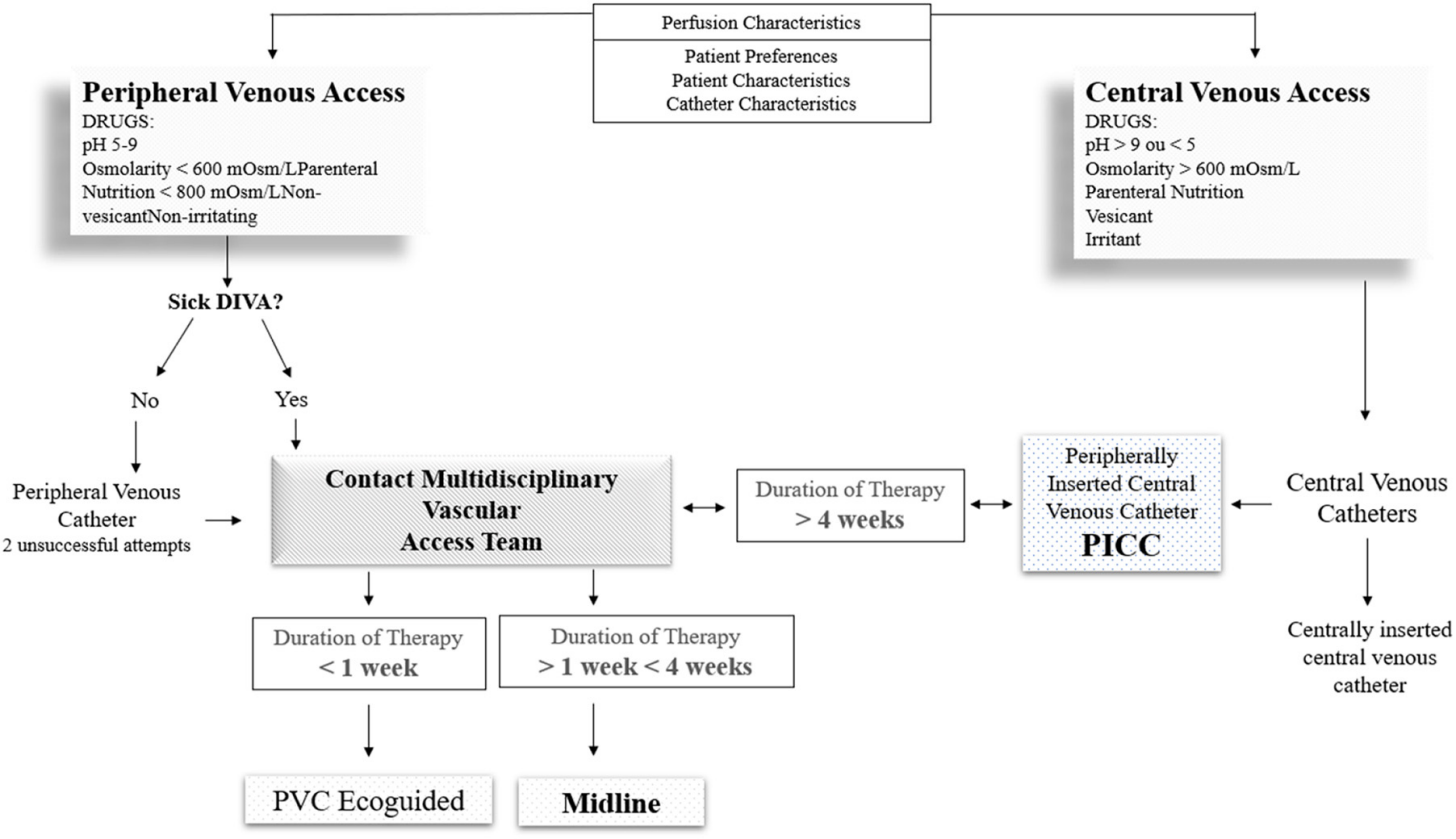
Flowchart of the actuation protocol.

In Portugal, studies report the need for two to eight puncture attempts before a successful insertion of the peripheral venous catheter, in 19.4 %–23.7 % of adult hospitalized patients [8]. This number is unacceptably high and contradicts current standards of care (Figure 1).

The Infusion Nurses Society (INS), in the 2016 Infusion Therapy Practice Standards document, is permissible up to four attempts at peripheral venous catheter placement, specifying two attempts per practitioner, not exceeding four punctures, since multiple attempts cause pain, procrastinate treatment, limit future vascular access, increase cost, and increase the risk of complications [5]. These data remind us that the selection of different devices must be careful and the need to have professionals prepared for the evaluation and insertion of echo-guided catheters is imminent. It is essential to choose a venous access device that prioritizes health and preservation of venous heritage. In a hospital context, the choice for venous access inevitably falls on short-term catheters, such as peripheral venous catheters, but in certain therapeutic situations it is appropriate to use centrally inserted catheters or short/medium-duration peripherally inserted devices, such as PICC (peripherally inserted central venous catheters) and *midline* [9]. In this sense, Recent guidelines advocate for the creation of specialized VATs to improve success rates, reduce complications, and enhance patient and professional satisfaction [8].

The argument for the insertion of a catheter by a specialized team is the service of best practices, and it is a consistent, experienced and qualified approach, as they reveal high levels of knowledge and confidence of the professional, built based on experience and procedural competence. There are models of action of the teams that focus only on the insertion of the catheter, and other models that include maintenance care, which may include clinical interventions, such as replacement of dressings and daily evaluation for possible removal. However, even though the models that the teams have as their goal limited only to insertion, there are reports of better results for the success of the first insertion [10].

Therefore, we carried out this study, divided into two parts: In the first, we sought to understand the importance of implementing a multidisciplinary team of vascular accesses and to diagnose the need for the existence of an action protocol, identifying the interventions that should be protocoled, from the perspective of the professionals; in the second we sought to validate the interventions of a consensus-based protocol of action of a multidisciplinary team of vascular accesses.

## Methods

A mixed-method study was conducted, combining a qualitative exploratory phase and a quantitative Delphi phase.

The qualitative aspect of this research work, the pre-study, aims to understand the importance of implementing a multidisciplinary team of vascular accesses from the perspective of professionals and to diagnose the need for the existence of an action protocol, identifying the interventions that should be protocoled. In this regard, for the definition of the sample we established the inclusion criteria as being healthcare professionals, nurses or doctors, who work in services with the possibility of contacting a multidisciplinary vascular access team. The first phase (qualitative) was developed with 20 participants (18 nurses, 2 physicians) who were given a semi-structured interview consisting of two parts: The first part referring to the characterization of the sample, consisting of some questions such as: age, gender, health professional, length of professional experience and area of expertise, and the second consisting of issues related to the importance of implementing a multidisciplinary vascular access team and diagnosing the need for the existence of an action protocol, identifying the interventions that should be protocolled. Qualitative data were analyzed through content analysis, according to the methodology proposed by por Bardin; Almeida et al. [11], 12] which are organized into three phases: i) pre-analysis (floating reading of the responses and redefinition of the objectives) ii) exploration of the material (creation of categories) and iii) treatment of the results, inferences, and interpretation. After analysis by the research team, the coding achieved total agreement.

The 2nd phase of the study aimed to validate the interventions of a consensus-based protocol of action of a multidisciplinary team of vascular accesses. For this purpose, we use the Delphi Method. The following inclusion criteria were defined for the selection of experts for the Delphi panel: being a doctor or nurse, having training in ultrasound-guided vascular access, having professional experience in the insertion and maintenance of devices, particularly PICC lines and midlines, and agreeing to participate in the study. The Delphi questionnaires were divided into three parts and preceded by a brief introduction, outlining the study’s objectives, its organization, and the way to fill it out. The first part of the questionnaire corresponds to the characterization of the experts, the second part to the interventions proposed at each stage of the consensus-based protocol, and the third part to the experts’ general suggestions. In order to assess the level of agreement or disagreement with each of the proposed interventions, we used a Likert scale. In this way, the scale had points numbered from one to five, which correspond respectively to strongly disagree; disagree; indifferent (or neutral); agree; and strongly agree.

Quantitative data were analyzed using descriptive statistics in SPSS 22, with consensus defined as ≥75 % agreement. Respecting the Delphi technique, we defined the systematic and staggered sending of questionnaires to experts, promoting controlled feedback on the opinions expressed and the collection of new opinions, carrying out the necessary number of rounds until the defined consensus is achieved. This phase was developed from a panel of 20 experts (18 nurses, 2 physicians) with experience in ultrasound-guided vascular access.

## Results

In the qualitative study we obtained important data on the possible benefits of implementing a VAT team; criteria for activating the VAT team; the actions that must be filed.

Thus, the main advantages of the performance of a team are VAT fewer punctures, fewer complications, improved patient and staff satisfaction, and reduced hospital stay. Regarding the criteria for activating the team, the main reason was the classification of patients with difficult venous access. Finally, in relation to the actions to be protocolized, the following categories were identified: evaluation of the peripheral venous network (through the A-DIVA scale), establishment of a maximum number of puncture attempts and Device Selection Criteria considering the characteristics of the catheter, the characteristics and duration of treatment, the preference and characteristics of the patient.

This phase allowed us to confirm the importance of the implementation of the VAT team, to know some gaps and the interventions that need to be protocoled, which is fundamental to develop effective protocols and procedures, ensuring a comprehensive and high-quality approach to vascular access management. It thus provided valuable insights that can guide the development of more effective and patient-centred clinical protocols and practices and, therefore, fundamental for the second phase of the study.

After the qualitative aspect of the pre-study, we started the study, properly speaking, for the elaboration of the respective protocol, with the constitution of a panel of experts who analyzed the agreement of the interventions to be included in a protocol of action of the multidisciplinary team of vascular accesses.

The age of the experts ranged from 29 to 45 years of age, with a mean of 37.30 years; mostly female. From a professional point of view, 18 are nurses and 2 are doctors; The average length of professional practice was 15.15 years, ranging from a minimum of 7 years to a maximum of 23 years. Most of the experts work in the intensive medicine service (75 %), followed by 10 % of professionals working exclusively in a multidisciplinary team of vascular accesses. All experts have training and experience in guided access.

Questionnaires were made available with the criteria and actions to be placed in a protocol (Table 1). Two rounds were carried out to obtain consensus and validation of the experts on the interventions that should be included in the protocol of action of a multidisciplinary team of vascular accesses.

**Table 1:** Results of the quantitative analysis of the agreement of the interventions.

Interventions	Agreement											
	1		2		3		4		5		Total	
	N	%	N	%	N	%	N	%	N	%	N	%
1. MODIFIED A-DIVA SCALE												
1.1 Use of the modified A-DIVA scale	0	0.0	0	0.0		0.0	0	0.0	20	**100,0**	20	100.0
1.2 Activation of the team in the diagnosis of a DIVA patient	0	0.0	0	0.0		0.0	0	0.0	20	**100,0**	20	100.0
2. Puncture attempts, before team activation												
2.1 Maximum two punches	0	0.0	0	0.0	0	0.0	0	0.0	20	**100,0**	20	100.0
2.2 Four attempts, two for each nurse	18	90.0	2	10.0	0	0.0	0	0.0	0	0.0	20	100.0
3. Selection criteria (patient characteristics, patient preferences, duration of therapy, perfusion characteristics, and catheter characteristics)	0	0.0	0	0.0	0	0.0	0	0.0	20	**100,0**	20	100.0
4. Characteristics of peripheral venous infusion:	0	0.0	0	0.0	0	0.0	0	0.0	20	**100,0**	20	100.0
– Osmolarity<600 mOsm/L,												
– pH between 5 and 9,												
– Parenteral nutrition with osmolarity<800 mOsm/L, – Drugs or solutions that are not associated with epithelial damage of irritating, vesicant, and necrotizing potential												
5. Characteristics of central venous infusion: – pH>9 or<5; – Osmolarity>600 mOsm/kg; – Vesicant and irritant medication. – Parenteral nutrition.	0	0.0	0	0.0	0	0.0	0	0.0	20	**100,0**	20	100.0
6. Duration of therapy – peripheral venous catheter												
6.1 Intravenous treatment less than 7 days	0	0.0	0	0.0	0	0.0	0	0.0	20	**100,0**	20	100.0
6.2 Intravenous treatment less than 10 days	18	90.0	2	10.0	0	0.0	0	0.0	0	**0.0**	20	100.0
7. Duration of therapy – medium lines, greater than 1 week, but less than 4 weeks	0	0.0	0	0.0	0	0.0	0	0.0	20	**100,0**	20	100.0
8. Duration of therapy – CVC, greater than 4 weeks	0	0.0	0	0.0	0	0.0	0	0.0	20	**100,0**	20	100.0
9. CVP removal – no catheter should be removed solely based on length of stay	0	0.0	0	0.0	0	0.0	0	0.0	20	**100,0**	20	100.0

N=20; 1-a strongly disagree; 2-disagree; 3-indifferent (or neutral); 4-agree; 5-strongly agree. A value of 100 % corresponds to the number of responses obtained in maximum agreement.

Analyzing the data presented, we can see that there is agreement in the opinion of the experts. Regarding the use of the modified A-DIVA scale, all experts agree with its use as a criterion for team activation.

When we are dealing with a non-AVD patient, according to the experts, the nurse responsible for the patient can perform a maximum of two puncture attempts. A patient who needs more than two puncture attempts is considered a patient with difficult venous accesses and for this reason the multidisciplinary team of vascular accesses should be activated to preserve their venous network.

Regarding the questions of the characteristics of the therapy, whether peripheral or central, they also obtained 100 % agreement.

Asked about the selection criteria, which include patient characteristics, patient preferences, duration of therapy, perfusion characteristics and catheter characteristics, all experts agree.

As we have seen in the theoretical framework, some authors advocate that the peripheral venous catheter is indicated for a treatment of less than seven days, and our experts agree, and discard the hypothesis that a peripheral venous catheter is indicated for a treatment of up to ten days.

The twenty experts, with a percentage of 100 %, agreed that midlines are indicated for a duration of therapy of more than one week, but less than four weeks, and central venous catheters more than four weeks.

The Delphi phase reached 100 % consensus on key points:

– Use of the modified A-DIVA scale for early DIVA detection;
– Maximum of two punctures before VAT activation;
– Individualized device selection based on patient characteristics, preferences, treatment duration, and therapy type;
– Clear criteria distinguishing peripheral from central access.

## Protocol for the multidisciplinary team of vascular accesses

With the construction of the protocol for the multidisciplinary team of vascular accesses, the expected result is the standardization of its performance and in this sense a flowchart was prepared that compiled all the interventions validated by the experts.

The action protocol was developed based on the validation of interventions by a panel of experts, resulting in the construction of a flowchart aimed at guiding healthcare professionals in selecting the most appropriate vascular access device.

The decision-making process is guided by criteria such as:

– Patient preferences and characteristics;
– Perfusion characteristics (osmolarity, pH, vesicancy, type of solution);
– Type and expected duration of intravenous therapy;
– Assessment of peripheral venous access using the Modified A-DIVA Scale.

Medications with an osmolarity below 600 mOsm/L, pH between 5 and 9, parenteral nutrition with osmolarity below 800 mOsm/L, and that are non-vesicant and non-irritant, are compatible with peripheral venous access, using a Peripheral Venous Catheter (PVC) or a Midline catheter. On the other hand, medications with pH values outside these ranges, osmolarity above 600 mOsm/L, vesicant or irritant properties, and concentrated parenteral nutrition require central venous access, such as a Peripherally Inserted Central Catheter (PICC) or a Centrally Inserted Central Venous Catheter (CVC).

The flowchart (Protocol for the Multidisciplinary Vascular Access Team) represents an objective clinical tool that guides clinical reasoning, standardizes practice, and supports evidence-based decision-making. Additionally, it serves as a guide for activating the specialized team, promoting a collaborative approach among different hospital departments.

This systematization enhances the efficiency of care, reduces the risk of complications, minimizes the number of unnecessary punctures, and increases satisfaction among both healthcare professionals and patients.

## Discussion

The formation of specialized vascular access teams has been a recommended strategy; the selection of different devices must be careful, and the need to have professionals trained in the evaluation and insertion of ultrasound-guided catheters is imminent, as is the implementation of validated protocols that support their use [5], 8]. The pre-study resulted in the participants’ opinion that the implementation of a VAT team can be important in reducing the number of punctures, their complications and thus improving patient satisfaction. These results confirm what has been pointed out in other studies [10], [11], [12], [13], [14] that refer to the specialized knowledge and experience of team members as a key factor for fewer complications, better success rates and greater overall patient satisfaction. This study also highlights the importance of establishing a standardized set of actions that allows the maximization of the use of a team with these characteristics, thus valuing the objective of the researchers to validate an action protocol.

The final study resulted in the fundamental points for a protocol, of which we highlight:

– Use of the modified A-DIVA scale for early DIVA detection; Performing an adequate assessment of the peripheral intravenous network of patients is a fundamental step in clinical practice. The A-Diva scale is, in the opinion of experts, a valuable tool for identifying patients at risk of difficult intravenous access, confirming what has already been pointed out in several studies [15], 16].
– Maximum of two punctures before VAT activation; This intervention promotes a culture of vascular care that values the integrity of the venous heritage, and in this line of thought, experts agree the recommendations of the literature [5], of a maximum of two attempts prior to the activation of the multidisciplinary team of vascular accesses, justifiable by the preservation of the peripheral venous network.
– Individualized device selection based on patient characteristics, preferences, treatment duration, and therapy type; The choice of the most appropriate vascular access device for our patient is crucial to ensure safe and effective treatment. Your selection should follow the following criteria: patient characteristics, patient preferences, duration of therapy, perfusion characteristics and catheter characteristics

In this way they indicate that safe and reliable venous access is essential, selecting the most appropriate device for the patient’s needs is essential, since the integrity of the vessel will be preserved and the person’s suffering minimized [17].

– Clear criteria for distinguishing peripheral access from central access. In a hospital context, the choice for venous access inevitably falls on short-term catheters, such as peripheral venous catheters, but in certain therapeutic situations it is appropriate to use centrally inserted catheters or short/medium-duration peripherally inserted devices, such as PICC (peripherally inserted central venous catheters) and midline [9]. Nurses must make sure that their patients have the most appropriate vascular access device and for this, these professionals must know the various devices and ensure safe and effective treatment.

The implementation of VATs, guided by a consensus-based protocol, addresses a critical need in vascular access management. When implemented, such a protocol has the potential to improve success rates and reduce complications, as suggested by existing literature and expert opinion [14], [15], [16], [17]. The use of ultrasound-guided techniques and the integration of the A-DIVA scale are particularly relevant innovations, promoting the preservation of venous capital and reducing healthcare costs. Importantly, the study fills a research gap in the Portuguese context, offering a replicable model for other healthcare institutions.

This study has some limitations, namely: the panel may be considered small and not very diverse (only 2 doctors, 75 % from the ICU, only 10 % currently involved in VATs) and the fact that there were only 2 rounds may not capture the full evolution of the consensus. On the other hand, it might be important to introduce the representativeness of the pharmacist group on the expert panel. In the future, we intend to develop new research focused on the implementation of this protocol in clinical settings and on evaluating its impact on insertion success, complication rates, cost-effectiveness, and patient satisfaction.

## Conclusions

This study developed and validated a VAT consensus-based protocol, ensuring evidence-based, standardized care. The protocol includes DIVA assessment, device selection, and activation criteria, contributing to improved clinical outcomes, cost efficiency, and patient satisfaction.

The protocol was built upon the latest international guidelines and validated by expert consensus, ensuring a structured and consistent approach to vascular access care.

When implemented, the work contributes significantly to improving the quality and safety of care, not only for the implementing team but also by offering a replicable model for other healthcare units. The incorporation of devices such as PICCs and midlines – highlighted throughout the theoretical framework – is identified as having a positive impact on clinical practice, offering safer and more effective vascular access solutions focused on patient care.

Given the critical importance of intravenous therapy in nursing practice, the experts in our study emphasize the importance of continuing professional education, the development of organized, protocol-based nursing interventions focused on preserving venous heritage and the systematic management of vascular access devices.

A key limitation identified is the limited existing literature and the lack of advanced training in ultrasound-guided vascular access in basic nursing education. This highlights the need to expand training programs to include specialized and advanced skills.

The proposed flowchart promotes a vascular care culture that values venous preservation and encourages overcoming resistance to change through education and empowerment of healthcare professionals.

In conclusion, this consensus-based protocol constitutes a valuable contribution to the systematization of critical thinking and to the structuring of the nurse’s autonomous intervention, with a real and effective impact on the quality of care provided to the user.
